# Supplementary material for: Systematic evaluation and meta-analysis of the prognosis of down-staging human papillomavirus (HPV) positive oropharyngeal squamous cell carcinoma using cetuximab combined with radiotherapy instead of cisplatin combined with radiotherapy
Source: PeerJ. 2024 May 20;12:e17391. doi: 10.7717/peerj.17391 (PMC11114112; doi:10.7717/peerj.17391)
Supplement: Supplemental Information 3 [file peerj-12-17391-s003.docx]

1. The rationale for conducting the meta-analysis.

D. Rischin et al. 2021 and J. Huanget al. (a meta-analysis of 31 retrospective and some prospective studies) as landmark trials in downstaging both concluded that using cetuximab instead of cisplatin in combination with radiotherapy significantly improved patient survival, although it did not reduce its toxic side effects. However, in subsequent trials conducted by multinational research groups, however, the opposite conclusion was reached: the incidence of adverse events was not significantly different between the two treatment groups, and the overall survival of patients was significantly reduced in both cases. Therefore, it is necessary to clarify the efficacy and safety of cetuximab combined with radiotherapy regimen compared with conventional cisplatin combined with radiotherapy regimen for HPV^+^ OPSCC and to conduct systematic evaluation and Meta-analysis.

1. The contribution that the meta-analysis makes to knowledge in light of previously published related reports, including other meta-analyses and systematic reviews.

In this study, by including five randomized controlled trials in the literature, all of which were meta-analyses of cetuximab combination radiotherapy versus cisplatin combination radiotherapy regimens for the treatment of patients with HPV^+^ OPSCC, it was clarified that cisplatin combination radiotherapy continues to serve as the gold standard for HPV^+^ OPSCC, and further subgroup analyses revealed that patients with low-risk HPV^+^ OPSCC with a smoking history of ≤10 packs/year by the 7^th^ edition of the AJCC/UICC criteria and without the involvement of Low-risk HPV^+^ OPSCC patients with non-pharyngeal tumors with lymphatic metastases under the 7th edition of the AJCC/UICC criteria, using cetuximab instead of cisplatin in combination with radiotherapy, had similar survival outcomes to the traditional regimen using cisplatin. This may be an alternative downstaging strategy after carboplatin/5-fluorouracil for patients who fit this profile and are also intolerant to cisplatin, especially if they are allergic to platinum-based agents.

M. Buglione, M. Maddalo, R. Corvo, L. Pirtoli, F. Paiar, L. Lastrucci, et al.Subgroup analysis according to human papillomavirus status and tumor site of a randomized phase II trial comparing cetuximab and cisplatin combined with radiation therapy for locally advanced head and neck cancer Int [J] Radiat Oncol Biol Phys, 97 (2017), pp. 462-472

M.L. Gillison, A.M. Trotti, J. Harris, A. Eisbruch, P.M. Harari, D.J. Adelstein, et al.Radiotherapy plus cetuximab or cisplatin in human papillomavirus-positive oropharyngeal cancer (NRG Oncology RTOG 1016): a randomised, multicentre, non-inferiority trial Lancet, 393 (2019), pp. 40-50

H. Mehanna, M. Robinson, A. Hartley, A. Kong, B. Foran, T. Fulton-Lieuw, et al.Radiotherapy plus cisplatin or cetuximab in low-risk human papillomavirus-positive oropharyngeal cancer (De-ESCALaTE HPV): an open-label randomised controlled phase 3 trial Lancet, 393 (2019), pp. 51-60

M. Gebre-Medhin, E. Brun, P. Engstrom, H. Haugen Cange, L. Hammarstedt-Nordenvall, J. Reizenstein, et al. ARTSCAN III: a randomized phase III study comparing chemoradiotherapy with cisplatin versus cetuximab in patients with locoregionally advanced head and neck squamous cell cancer [J] Clin Oncol, 39 (2021), pp. 38-47

Curran D, Giralt J, Harari PM, et al. Quality of life in head and neck cancer patients after treatment with high-dose radiotherapy alone or in combination with cetuximab. [J] Clin Oncol 2007;25:2191–7.
