# Supplementary material for: Systematic evaluation and meta-analysis of the prognosis of down-staging human papillomavirus (HPV) positive oropharyngeal squamous cell carcinoma using cetuximab combined with radiotherapy instead of cisplatin combined with radiotherapy
Source: PeerJ. 2024 May 20;12:e17391. doi: 10.7717/peerj.17391 (PMC11114112; doi:10.7717/peerj.17391)
Supplement: Supplemental Information 4 [file peerj-12-17391-s004.docx]

**A**

**
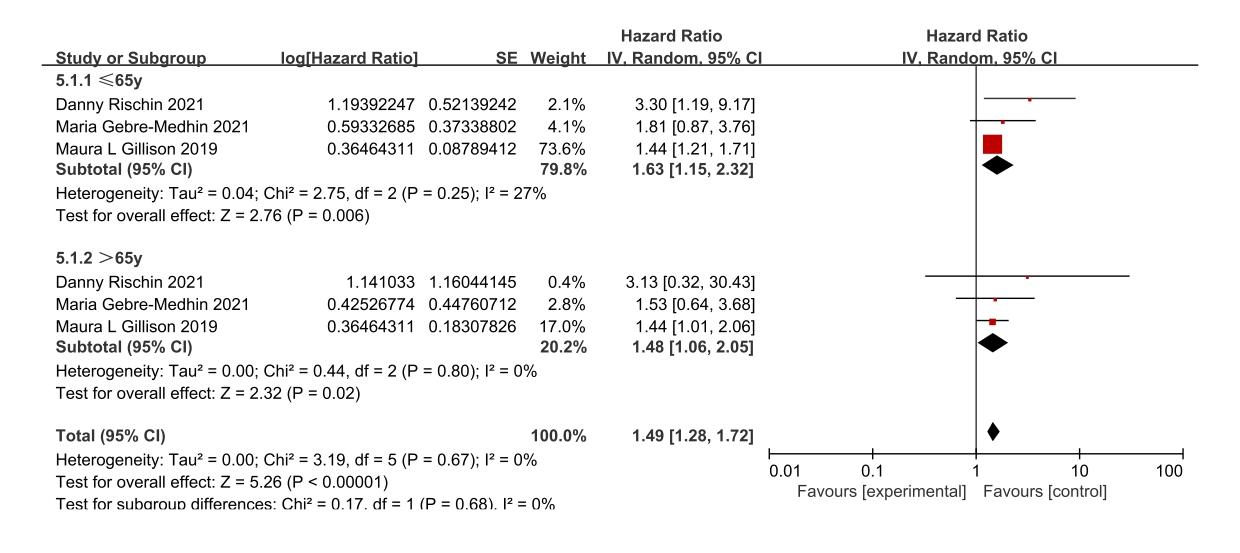
**

**B**


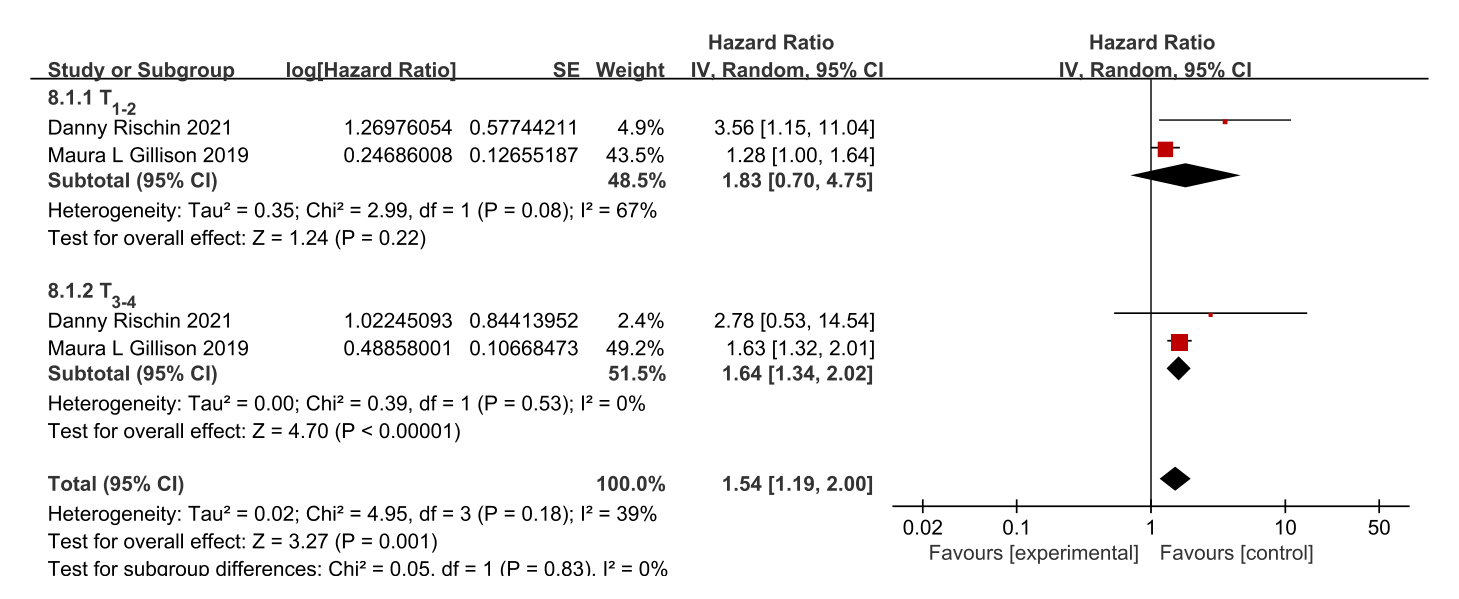


**Supplementary figure 1** (A) Forest plot of overall survival subgroup analysis based on age of patients. (B) Forest plot of overall survival subgroup analysis based on primary tumor stage T of patients.
