# Supplementary material for: Systematic evaluation and meta-analysis of the prognosis of down-staging human papillomavirus (HPV) positive oropharyngeal squamous cell carcinoma using cetuximab combined with radiotherapy instead of cisplatin combined with radiotherapy
Source: PeerJ. 2024 May 20;12:e17391. doi: 10.7717/peerj.17391 (PMC11114112; doi:10.7717/peerj.17391)
Supplement: Supplemental Information 6 [file peerj-12-17391-s006.docx]

**A**


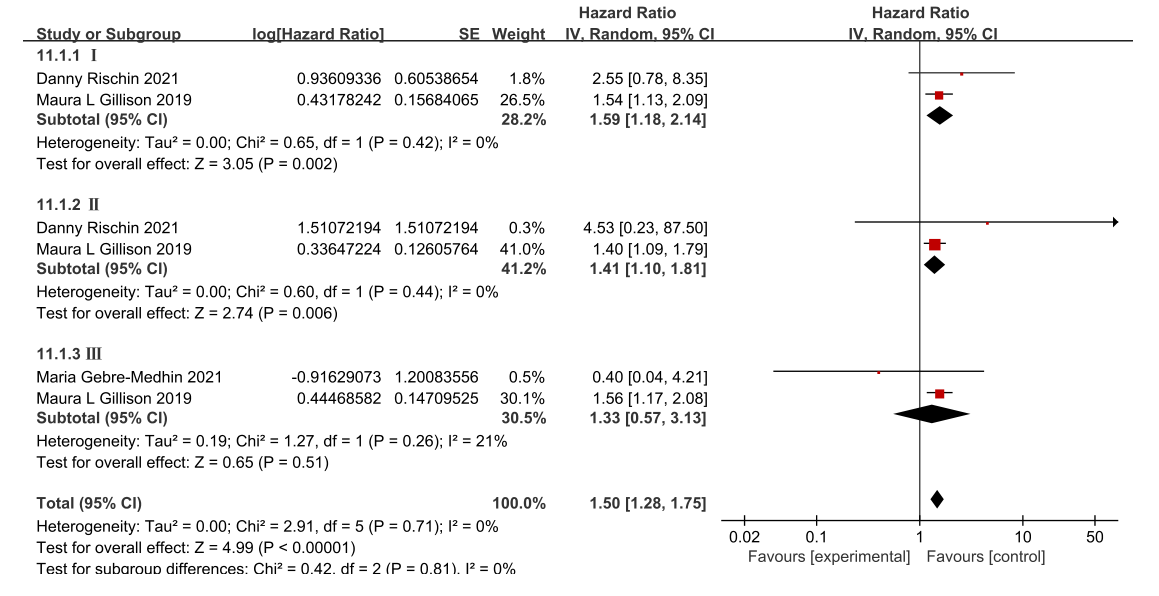


**B**


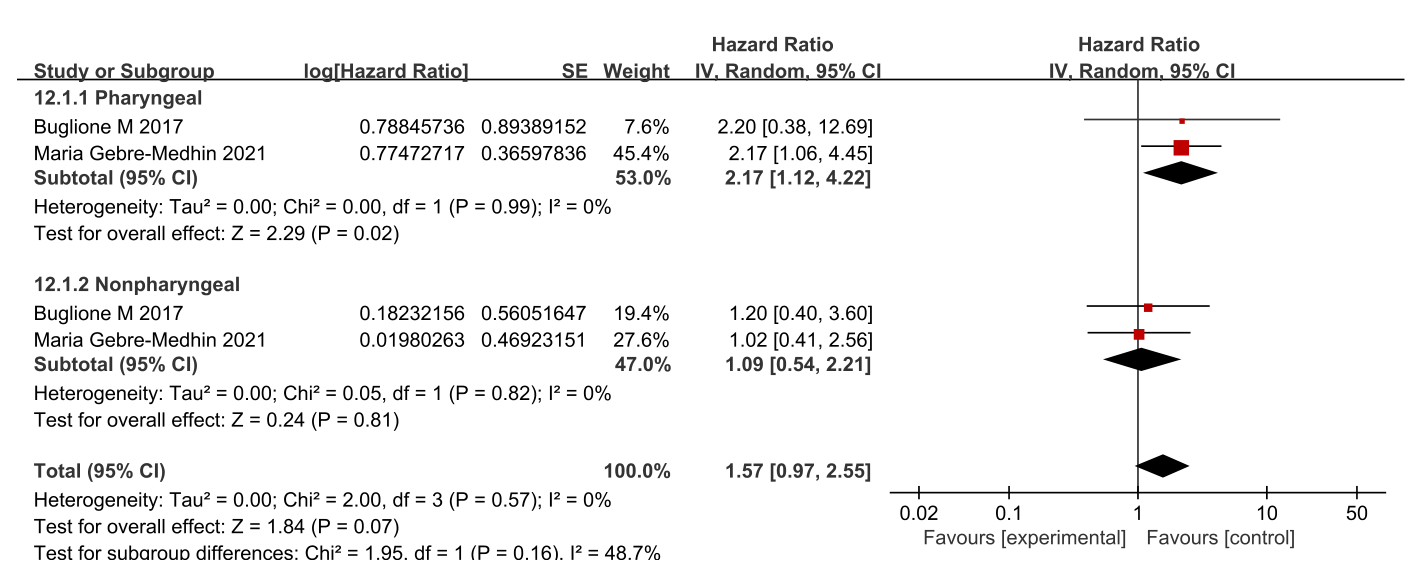


**Supplementary figure 3** (A) Forest plots for OS subgroup analysis according to the 8^th^ edition UICC/AJCC definition of primary tumor overall stage.（B）Forest plots were analyzed for subgroups of OS according to primary tumor site.
