# Supplementary material for: Systematic evaluation and meta-analysis of the prognosis of down-staging human papillomavirus (HPV) positive oropharyngeal squamous cell carcinoma using cetuximab combined with radiotherapy instead of cisplatin combined with radiotherapy
Source: PeerJ. 2024 May 20;12:e17391. doi: 10.7717/peerj.17391 (PMC11114112; doi:10.7717/peerj.17391)
Supplement: Supplemental Information 7 [file peerj-12-17391-s007.docx]

**A**


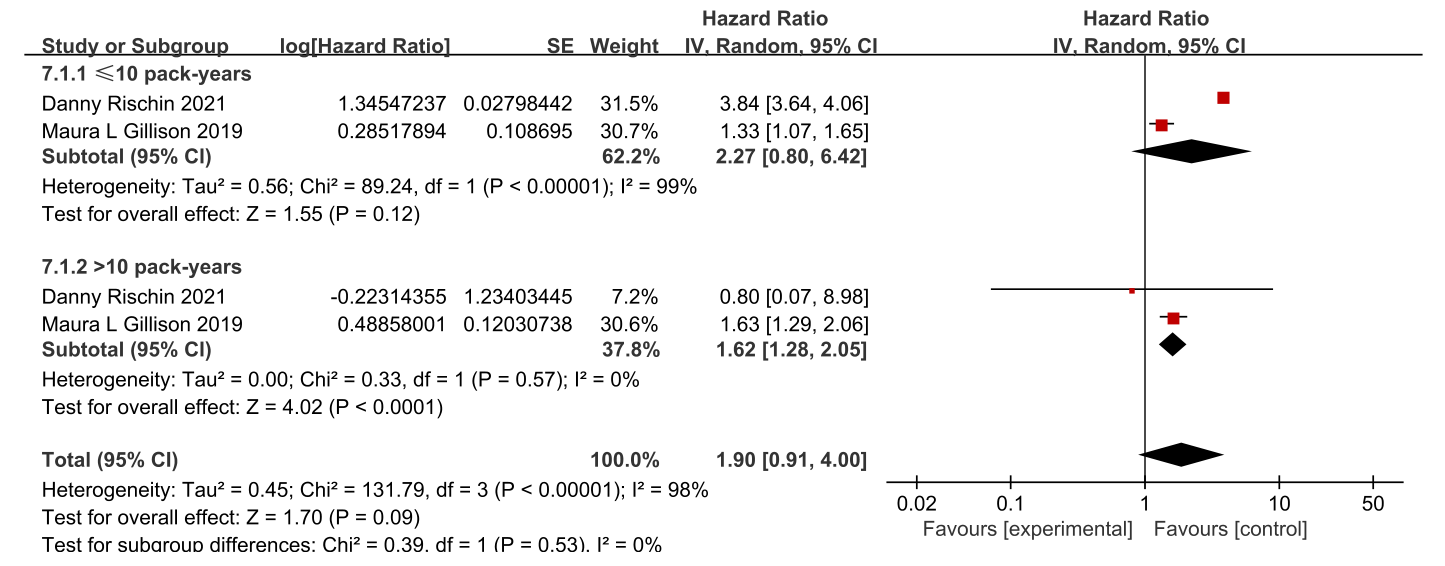


**B**

**
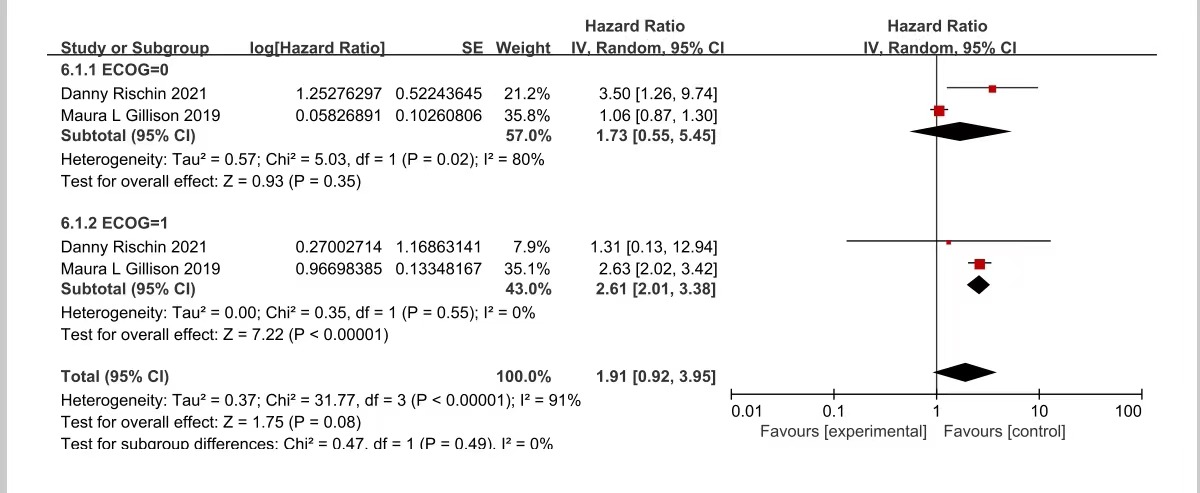
**

**Supplementary figure 4** (A) Forest plot of OS subgroup analysis according to the smoking history of patients. (B) Forest plot of OS subgroup analysis based on ECOG score of patients.
